# Motives and considerations regarding PGT in couples carrying a structural chromosomal abnormality: a qualitative exploration

**DOI:** 10.1007/s10815-020-01810-w

**Published:** 2020-05-16

**Authors:** G. De Krom, Y. Severijns, W. L. Vlieg, Y. H. J. M. Arens, R. J. T. Van Golde, C. E. M. De Die-Smulders, L. A. D. M. Van Osch

**Affiliations:** 1 Department of Obstetrics & Gynaecology, Maastricht University Medical Centre +, Maastricht, The Netherlands; 2 School for Oncology and Developmental Biology, GROW, Maastricht University, Maastricht, The Netherlands; 3 Department of Health Promotion/CAPHRI, Maastricht University, PO Box 616, 6200 MD Maastricht, The Netherlands; 4 Department of Clinical Genetics, Maastricht University Medical Centre +, Maastricht, The Netherlands

**Keywords:** Preimplantation genetic testing, Structural chromosomal abnormalities, Reproductive decision-making, Qualitative analysis

## Abstract

**Purpose:**

This study aims to describe the motives and considerations of couples carrying a structural chromosomal abnormality deciding on preimplantation genetic testing (PGT).

**Methods:**

A qualitative exploratory study was conducted using semi-structured dyadic interviews with 13 couples (*N* = 26) carrying a structural chromosomal abnormality. All couples had an informative consultation in our PGT centre in the Netherlands.

**Results:**

Almost all couples considered PGT or natural conception combined with prenatal diagnosis (PND) as the only two reproductive options. Among several considerations mentioned, the majority indicated that the wish to increase the chance of a successful pregnancy was the most important motive to opt for PGT. All couples who opted for PGT had first tried to conceive spontaneously and entered the PGT programme because of their adverse experiences during these attempts (infertility, recurrent miscarriage, termination of pregnancy, birth of an affected child). Couples that refrained from PGT were of advanced maternal age and expressed the long trajectory of PGT as the main reason to refrain. If conceiving spontaneously would not lead to an ongoing pregnancy, these couples also indicated that they would use PGT.

**Conclusion:**

This study shows that couples carrying a structural chromosomal abnormality consider PGT and spontaneous conception with PND as relevant reproductive options. They are looking for the option that is in their opinion the fastest way to establish a successful pregnancy. Information on the perceived pros and cons of PGT or spontaneous conception in these couples can help to optimize counselling and psychological support during the decision-making process.

## Introduction

Individuals carrying a balanced structural chromosomal abnormality are at risk for infertility and may produce gametes with an unbalanced karyotype. An unbalanced conceptus may lead to failure to implant; early or late miscarriage; or more seldom an ongoing pregnancy of a foetus with an unbalanced karyotype resulting in physical or mental disabilities in the child [1, 2]. The risk for an unbalanced karyotype in offspring depends on the chromosomes involved and the type of chromosomal abnormality [3]. Prenatal diagnosis is offered to couples carrying a chromosomal abnormality. PND is indicated during an ongoing pregnancy to allow for termination of pregnancy (TOP) in case of an unbalanced karyotype. Preimplantation genetic testing (PGT) is an alternative to natural conception with or without PND for couples in whom one of the partners carries a structural chromosomal abnormality. [4–7]. In PGT, embryos derived from in vitro fertilization (IVF) were genetically tested using fluorescence in situ hybridization (FISH), array comparative genomic hybridization (CGH) or comprehensive chromosome screening [1, 8]. Only embryos with a normal or balanced karyotype are eligible for transfer in the uterus. In the Netherlands, the costs for PGT as well as PND are covered by the health insurance system [9]. Alternatives to PND or PGT for couples genetically at risk are the use of donor gametes, adoption or remaining childless.

Previous research focused especially on the decision-making process regarding PGT in monogenic disorders and it might be that motives and considerations between these groups are different. Couples wanting to reproduce in whom one of the partners carries a structural chromosomal abnormality differ in multiple ways from couples with other PGT indications. Couples with a monogenic disorder have a 25 or 50% risk of affected offspring. Couples carrying a structural chromosomal abnormality generally have a low risk of an ongoing unbalanced conceptus [1]. However, they can experience difficulties getting pregnant due to infertility or failure to implant or face an increased risk of a miscarriage, sometimes followed by medication or surgical abortion [10]. Therefore, it is likely that in couples carrying a structural chromosomal abnormality, the decision-making process regarding PGT or natural conception with PND is characterized by different considerations compared with that in couples with monogenic conditions [11].

One would expect that the reproductive and obstetric history in this patient group is a main factor influencing the decision on PGT in these couples. However, in a previous retrospective study in recurrent miscarriage couples carrying a structural chromosomal abnormality, we found that there is no difference in obstetric history (e.g. number of miscarriages, TOP or affected children born) of couples who opt for PGT compared with those that decline PGT after extensive genetic counselling [12]. In order to optimize counselling, it is important to understand couples’ thoughts when making a reproductive choice. This raised the question of what motives and considerations these couples have when making a decision on whether or not to pursue PGT.

This study reports the qualitative analysis of the reproductive decision-making process among couples carrying a structural chromosomal abnormality.

## Material and method

### Recruitment of couples

Couples in whom one of the partners carries a structural chromosomal abnormality were eligible for participation in the study if they had an informative consultation concerning PGT and other reproductive options, mainly natural conception and PND. Verbal and written information was given on the PGT procedure, including the IVF-intracytoplasmic sperm injection (ICSI) treatment, success rates, possible complications, risks of misdiagnosis and health of children born after PGT. Counselling was performed at Maastricht University Medical Centrum+ (MUMC+) and 1 couple in the Amsterdam University Medical Centers (Amsterdam UMC), both hospitals participating in ‘PGT The Netherlands’, a national collaboration network for PGT in the Netherlands. The maximum interval between counselling and participating in the interview was 6 months.

Further inclusion criteria were as follows: age of both partners ≥ 18 years and full understanding of the Dutch language. Exclusion criteria were not meeting the criteria for PGT (e.g. medical reasons, severe physical or psychological illness) or IVF-ICSI (e.g. female age > 41, BMI > 35, FSH level > 15 IU/ml (in the early follicular phase)).

Recruitment of couples for the study took place after the informative PGT consultation. A letter of invitation, a patient information letter with informed consent forms for both partners and a return envelope was given to the couple directly after the informative PGT consultation, or was sent to couples’ home address. The investigators were available to answer questions regarding the study. The investigator contacted the couples who gave informed consent by telephone to plan the interview with both partners. Couples who did not return the informed consent forms within 2 weeks were contacted by telephone to answer any prevailing questions and collect reasons for non-participation. The consent form specifically stated that the researcher was independent from the treating physician(s), that the study team will guard the privacy of all participants and data will be treated confidentially and would only be used for researcher purposes. Additionally, the consent form stated that participation in this study will not influence participants’ medical treatment.

In the study period, 62 couples meeting the inclusion criteria had an informative PGT consultation. Purposive sampling was conducted to include a study population with variable obstetric history (no pregnancy, recurrent miscarriage, (in)fertile, TOP, affected child) and to include couples that chose PGT as well as couples that refrained from PGT after reproductive counselling. Out of the 62 eligible couples, 24 were invited to participate in the study.

### Procedure

Dyadic interviews were conducted with both partners simultaneously at their home or at MUMC+ and lasted between 30 and 100 min. Prior to the start of the interview, the participants were asked to complete a questionnaire about demographic characteristics (sex, age, level of education, marital status, level of income, ethnic group), IVF-ICSI indication because of infertility (yes/no) and reproductive history (e.g. number of miscarriages). All interviews were conducted by the same investigator (GK, female researcher and resident obstetrics and gynaecology, not part of the reproductive counselling staff) with knowledge of PGT. Semi-structured interview approach was applied, using an interview guide consisting of four main questions. Firstly, participants were asked about perceived advantages of PGT and natural conception with or without PND and secondly, they were asked about perceived disadvantages. Several cues (regarding medical, psychological, practical, ethical and financial aspects) were used to gather more information if necessary [11]. Subsequently, they were asked to indicate the most influential factor in their decision and their considerations regarding other reproductive options, such as remaining childless, adoption and the use of donor gametes. Finally, participants were asked how they experienced the decision-making process, with subquestions inquiring after, e.g. the moment of actual decision-making in relation to the informative consultation on PGT, the perceived need for support in their decision-making process and which persons had influenced their decision-making.

### Data preparation and analysis

All interviews were audio recorded and transcribed verbatim. The transcription did not include any personal data; confidentiality was guaranteed. The obtained data were analysed using the software program NVivo 10. Two researchers independently coded the data. In case of disagreement, a third researcher was consulted in order to reach consensus. Open coding of the data was followed by axial coding, organizing the data into segments based on keywords and concepts to form categories and identify major themes. During the last few interviews, no new considerations and motives were mentioned suggesting saturation of core themes.

## Results

### Couples’ characteristics

The participation rate was 54.2% (13/24). Reasons for non-participation are summarized in Table 1. Eight of 13 couples interviewed had decided to undergo PGT. Two couples decided to try to achieve a natural pregnancy. Three couples had started the PGT work-up but still continued trying to conceive naturally. The couples’ characteristics are summarized in Tables 2 and 3.

**Table 1:** Main reasons for non-participation (*n* = 11)

Reason	*n* (couples)
Not interested	3
Undergoing PGT treatment at the moment and want to fully focus on that	2
No response to the invitation letter; unable to establish contact	2
Experiencing a difficult time because of private circumstances	2
Unwillingness of one of the partners to participate in an interview	1
Lack of time	1

**Table 2:** Couples’ characteristics

	Reproductive choice		
	PGT (*n* = 8)	Natural conception (*n* = 2)	Both^a^ (*n* = 3)
Carrier partner (M/F)	7/1	1/1	0/3
Type of structural chromosomal abnormality			
o Reciprocal translocation (M/F)	3/0	1/1	0/2
o Robertsonian translocation (M/F)	4/0		0/1
o Pericentric inversion (M/F)	0/1		
Reason for karyotyping			
o Family history	3	1	
o Recurrent miscarriage	1		2
o Abnormality detected in previous pregnancy	2		1
o Affected child	2		
o Other; screening for malignancy		1	
Mean age (years) at the time of the interview (SD)			
o Male	31.4 (5.0)	32.0 (7.1)	38.0 (2.6)
o Female	29.0 (5.2)	30.5 (9.2)	34.3 (3.1)
Education^b^			
o Education low (M/F)	3/0		
o Education middle (M/F)	3/4	2/0	0/2
o Education high (M/F)	2/4	0/2	3/1
Occupation			
o Unemployed (M/F)	0/1	0/1	
o Part-time employment (M/F)	0/5	1/0	0/1
o Full-time employment (M/F)	8/2	1/1	3/2
Religion			
o Christianity (M/F)	1/1	1/1	0/1
o Not religious (M/F)	7/7	1/1	3/2

*PGT*, preimplantation genetic testing; *n*, number of couples

^a^PGT and natural conception at the same time

^b^Education was classified as low: pre-primary, primary and lower secondary education; middle: upper secondary education; and high: postsecondary education (Netherlands Central Bureau of Statistics, 2006)

**Table 3:** Couples’ reproductive history

Couples’ code	IVF-ICSI indication	Unaffected child (no.)	Live born affected child (no.)	Miscarriages (no.)	TOP/IUFD of the affected child (no.)	Pregnant at the time of interview	Reproductive choice
1	-	1	-	2	1 (TOP)	-	PGT
2	-	-	-	4	-	-	Both
3	Yes	-	-	-	-	-	PGT
4	-	-	-	4	-	-	PGT
5	-	1	-	4	-	Yes (8 weeks)	Both
6	Yes	-	-	-	-	-	PGT
7	Yes	-	-	-	-	-	PGT
8	-	1	-	2	1 (IUFD)	Yes (14 weeks)	Both
9	-	-	-	-	-	-	Natural conception
10	-	-	1 (trisomy 18)	-	-	-	PGT
11	-	-	-	-	-	-	Natural conception
12	-	1	-	1	2 (two times TOP)	-	PGT
13	Yes	-	1 (trisomy 21, after IVF)	-	-	-	PGT

*IVF*, in vitro fertilization; *ICSI*, intracytoplasmic sperm injection; *TOP*, termination of pregnancy; *IUFD*, intrauterine foetal death; *PGT*, preimplantation genetic testing

### General results

Almost all couples (11/13) considered PGT and natural conception combined with, PND as the only two acceptable reproductive options. Two couples did not explicitly mention whether natural conception with or without PND was acceptable for them. Most of the couples were aware of other options such as adoption and the use of donor gametes. Although some couples (10) briefly mentioned these options during the interview, only one couple seriously considered these options. They indicated that adoption and the use of donor gametes could become a possibility if PGT or natural conception with PND would be unsuccessful. All couples mentioned that remaining childless was not an option for them at this moment or in the near future.

Couples’ motives and considerations regarding PGT could be classified into physical, psychological, ethical and practical aspects (Table 4).

**Table 4:** Motives considered regarding PGT and natural conception with PND among couples carrying a structural chromosomal abnormality

	Preimplantation genetic testing—*natural conception with PND*	
	Motives to choose (*n*)	Motives to refrain (*n*)
Physical	Avoidance of miscarriages (8) Avoidance of TOP (4) Avoidance of ongoing pregnancy with unbalanced karyotype (6) Improving pregnancy chances by using ART (7) Avoidance of PND in case of an ongoing pregnancy (2) Healthy child (7) *Close monitoring of a pregnancy because of possibility of unbalanced conceptus and PND provided in pregnancy (1)*	Physical burden of IVF treatment (11) *Physical strain of recurrent miscarriage (7)* *Risk of an unbalanced ongoing pregnancy (6)*
Psychological	Avoidance of stress involving natural conception (5) Reassurance from beginning of pregnancy (5) Feel like having tried everything (2) *Preservation of romance and control regarding pregnancy (2)* *No interference in natural process of fertilization (1)*	Psychological strain emerging from success-related uncertainties during trajectory (6) Potential influence of hormone treatment on mood and emotions (5) Fear of disappointment if there is no success despite PGT (2) Loss of romance and control regarding pregnancy (4) Fear of negative reactions from environment (3) *Psychological burden when deciding on TOP in case of an unbalanced foetal karyotype (4)* *Psychological burden of recurrent miscarriage (1)* *Psychological burden when waiting for PND result (1)* *Fear every month of not getting pregnant (2)* *Feeling stress in early stage of pregnancy because of risk of unbalanced conceptus (4)* *Psychological strain emerging from uncertainties whether the embryo is affected or not (3)*
Moral/ethical	-	Interference in a natural process (3) Difficulty with possible disposal of rest embryo’s (1) *Feeling unable to cope with possible birth of an affected child in their family life (1)*
Practical	*Reasonable chance of achieving an ongoing balanced pregnancy when trying to conceive naturally for a longer period (5)* *Possible to start immediately, no long trajectory (2)* *Not necessary to invest time in hospital appointments (2)*	Moderate success rates (7) Relatively long duration of trajectory (7) Waiting time in trajectory (5) Frequent hospital appointments (5) Time investment in travelling to the PGT centre (12)

*N*, number of couples that considered this motive (non-correlated to decisiveness); *IVF*, in vitro fertilization; *ICSI*, intracytoplasmic sperm injection; *PGT*, preimplantation genetic testing; *TOP*, termination of pregnancy; *PND*, prenatal diagnosis

### Motives and considerations to opt for PGT

All couples who opted for PGT had tried to conceive naturally at first. They chose for PGT because of their experiences during these attempts as is shown in the following results.

The couples mentioned physical, practical and psychological motives to opt for PGT.

The main motive, which can be classified as physical and psychological, mentioned by the majority of participants (8/13) who opted for PGT, was to avoid miscarriages.

Female partner C2: …Only to protect myself from more frustrations and another miscarriage.

Two couples dealt with the termination of an unbalanced pregnancy and chose PGT to avoid this and two other couples who did not experience a TOP still mentioned preventing TOP as a perceived advantage of PGT.

Female partner C1: You have to go to a delivery room to give birth, which is quite something. You will not return happy.

Relatedly, the majority of the couples (7/13) indicated that a main physical motive to opt for PGT was their wish to increase the chance of a successful pregnancy. This especially applied to the infertile couples (4) wanting to increase their pregnancy chances using assisted reproductive techniques (ART). But also, some fertile couples (3/9) considered the fact that IVF-ICSI is needed for PGT as an advantage as they hope this will increase their pregnancy chances.

Female partner C4: Well, the success rate of IVF/PGT is higher, success rates are up to 15%; my natural chances are lower. So I rather first try IVF/PGT and maybe later if it does not succeed we will try naturally after all.

About half of the couples specifically mentioned that they wanted certainty that an unbalanced conceptus would be avoided.

Female partner C2: With PGT you can be sure that, if an embryo is transferred, it has a chance to grow. If you try to conceive naturally, there is this instant question whether it will be successful or not.

Two couples had a child with an unbalanced karyotype and wanted to avoid having another affected child.

Female partner C10: We want to be able to focus on the care that our daughter [with Edwards syndrome] needs. And it would be nice, if it is possible, to also have a few healthy children around.

A main psychological motive, mentioned by 5/13 couples, was the avoidance of stress during the waiting time for test results of PND. Furthermore, five couples mentioned the feeling of reassurance of avoiding an unbalanced pregnancy from the beginning of the pregnancy.

Male partner C9: The advantage [of PGT] is that we can select the embryo in an early stage before the beginning of pregnancy and choose the one without an unbalanced translocation.

Some couples indicated that they found it reassuring to know that PGT is available if other reproductive options fail (5 out of 13 couples). No practical motives to opt for PGT were mentioned.

### Motives and considerations to refrain from PGT

Couples mentioned several motives to refrain from PGT. The physical burden of the IVF treatment was perceived as an important disadvantage of PGT by the majority of couples (11/13). Five of the couples also mentioned the potential influence of the hormone treatment on the mood and emotional wellbeing of the female partners as a disadvantage of IVF treatment.

Practical considerations mentioned by most participants were the relatively long duration of the trajectory, waiting time to start with the trajectory, time investment in travelling to the PGT centre and frequent hospital appointments. However, most couples that opted for PGT or those that chose both PGT and natural conception added that these drawbacks of the PGT treatment did not significantly influence their reproductive choice. These couples were willing to cope with these practical issues and accepted these as part of the PGT treatment process.

Male partner C5: the biggest drawback [of PGT] is of course the hassle. You have to reschedule your agenda for a few weeks. And of course we would do anything for it, if you make this choice, that is not the issue. But I mean it has to be done [reschedule your agenda].

For the two couples that refrained from PGT, the long trajectory was a notable motive for their decision. They both added that the age of the female partner influenced their feeling of being ‘in a hurry’ to get pregnant. As the PGT trajectory takes a considerable amount of time, they hoped that trying to conceive naturally would lead to an ongoing pregnancy sooner. They furthermore indicated that if conceiving naturally would not lead to an ongoing pregnancy, they would consider trying PGT.

C9 male partner: It takes one year before you can actually start [PGT treatment] and another year before the treatment cycles are finished and you know if it succeeded. So the trajectory takes at least two years. And then with the age of [partner], she is 38 (…) we think we could first try ourselves [to conceive naturally], because we can start PGT till the age of 40.^1^

Half of the couples expect to experience a psychological strain during the PGT trajectory emerging from success-related uncertainties. A minority of couples (4/13) mention the loss of romance and control regarding pregnancy as drawbacks of PGT.

Male partner C7: I think the clinical aspect that it is necessary to handle your child wish in this way… You expect it to be something of us together at home. Now it will be a hospital affair.

Female partner C7: All of it is not that romantic.

### Making a reproductive choice

When asked within what time frame after the informative PGT consultation couples made their reproductive decision, the majority of couples indicate they made their decision within 24 h after reproductive counselling (4) or within 2 weeks (3).

Female partner of C12: Halfway through the consultation [informative consultation by a PGT specialist] we decided to go for it [PGT].

Only two couples took a longer time: 4 weeks and 12 months respectively. Four couples indicated that they already decided to pursue PGT before they had the informative PGT consultation. They mentioned that they had already gathered enough information on PGT, among others from the clinical geneticist that referred the couple to the PGT clinic, from their gynaecologist and from information on the Internet. They stated that they used the informative PGT consultation to gather more (practical) information on the treatment and to initiate the PGT process.

All couples indicated that the decision to opt for or refrain from PGT was made by the two of them together. The majority of couples discussed their choice with their family and friends, but this did not substantially influence the decision-making process. Two couples consulted a psychologist or social worker during the decision-making process. When asked if, in retrospect, they had felt the need for support from a psychologist during the decision-making process, only a minority of couples indicated that this would have been helpful.

## Discussion

This study provides a qualitative assessment of the decision-making process regarding PGT of couples in whom one of the partners carries a structural chromosomal abnormality.

There was a large overlap in motives and considerations to opt for or refrain from PGT between couples choosing PGT, natural conception with PND or both reproductive options.

The main motive to opt for PGT was that couples believe PGT will improve their chances of an ongoing pregnancy and thus shorten the time to (an ongoing) pregnancy of a healthy child in comparison with trying to conceive naturally. Other physical motives to opt for PGT are preventing of TOP or the birth of an affected child. This is in line with the rationale of offering PGT to these couples and with previous studies [13–15]. Additionally, PGT can lower the risk of miscarriages for translocation carriers to that of the population level [16].

An important motive to refrain from PGT is the expected physical and psychological burden of the IVF treatment. This is in line with previous studies among carriers and partners of carriers of a BRCA mutation who also indicate the psychological and physical burden of IVF (e.g. loss of romance, losing control as a couple) as main motives to refrain from PGT [9, 11].

It is well known that many couples indicate that the emotional burden of PGT and the IVF treatment entail is considerable [11, 17–19]. In our study, we found that half of the couples mention psychological strain from treatment-related uncertainties as a disadvantage of PGT. In addition, PGT for structural chromosomal abnormalities has limited success rates, with a delivery rate of 15% per oocyte retrieval [20, 21]. For some couples, this was seen as a drawback of PGT.

Our study design only included couples who visited a PGT clinic for reproductive counselling. This should be taken into account when interpreting the results as other reasons may be relevant for those who are not referred for or do not attend PGT counselling. All couples were well informed regarding PGT and underwent extensive genetic counselling before they participated in the study. This could imply that the couples in this study made a more deliberated decision as they were well able to weigh the pros and cons regarding PGT as they received extensive genetic counselling. Furthermore, we used dyadic interviews; i.e. both partners within the couple were interviewed simultaneously which may be of influence to the responses of the partners. However, joint interviewing has also several advantages as partners can comfort each other if one partner recounts painful experiences. Additionally, dyadic interviewing can provide insights into the shared experiences and meanings of the couple, which is less likely to achieve in individual interviews as reproductive decision-making is a shared experience [22].

In previous retrospective studies, the time between reproductive decision and participation in the study was often relatively long, leading to a risk of recall bias. In this study, we aimed to approach couples during or shortly after their decision-making process resulting in less risk of (recall) bias. Most couples were interviewed within 6 months after the genetic counselling on PGT, likely resulting in less risk of (recall) bias and a more complete overview of relevant considerations.

Previous studies on the reproductive decision-making process in couples who are at risk of transmitting a genetic disease indicate that reproductive decision-making is often a demanding and complex process in which multiple, sequential decisions are made and many factors are considered in order to reach a decision [11, 23, 24]. In a study from Derks-Smeets et al. (2014), couples with a BRCA mutation mentioned several significant advantages of PGT (e.g. protecting the child and family from the physical and psychological burden of the mutation, moral duty to protect their child) and many smaller disadvantages (e.g. the necessity of in vitro fertilization, perceived low chance of pregnancy by IVF/PGT) (10). A review on patient decision-making factors regarding PGT showed that one of the main reasons to refrain from PGT was the costs of this process [18, 25] However, in the Netherlands, health insurance companies cover the costs of the PGT treatment [11]. Therefore, none of the participants mentioned treatment costs as a main motive in this study. The amount and variety of considerations deliberated on seems to differ from our study, in which the main motives pertained to achieving an ongoing healthy pregnancy. Previous studies mainly focused on couples with monogenic disorders. These studies have shown that the time of a PGT treatment and the low pregnancy chances were important motives to refrain from PGT [11, 26]. Couples with a balanced rearrangement with a high risk of recurrent miscarriage usually have a low risk of an unbalanced ongoing pregnancy, which is in contrast with the risk of affected offspring in monogenic disorders [1]. The motives and considerations and extent of deliberation seem to be strongly dependent on the genetic condition PGT is considered for [27].

This study shows that couples carrying a structural chromosomal abnormality consider both PGT and natural conception with PND as personally acceptable reproductive options. Their main motive is achieving a successful pregnancy, with time to pregnancy as a main factor in their deliberation of reproductive options.

Motives are formed in response to couples’ reproductive history (e.g. history of miscarriages, TOP, affected child, infertility). Especially PGT is perceived as a way to avoid a conception that may result in a miscarriage [28]. Previous results showed that reproductive choices were not influenced by the reproductive history of translocation carriers [12]. Couples who experienced more unbalanced pregnancies were not more likely to opt for PGT than couples who did not experience unbalanced pregnancies. Another study on PGT, which included couples with different types of hereditary disorders, showed that the experience of a miscarriage increased the intention to opt for PGT. However, the actual method on how to achieve a successful pregnancy, PGT or spontaneous, seemed to be of less importance [29]. This could imply that the subjective way people experienced their reproductive history, as opposed to the objective reproductive history, is determinative in their decision regarding reproductive options. However, as stated before, one should consider that not all translocation carriers chose to have genetic counselling. Thus, the proportion of patients considering PGT as a reproductive option is smaller and these couples may have other motives to refrain from PGT.

In this study, couples who experienced a miscarriage opted for PGT because they wanted to prevent another miscarriage and PGT can lower the risk of miscarriage for translocation carriers. Understanding couples’ motives and considerations may help in genetic counselling and clinical care in these couples and may enable them to make a reproductive decision.

## Notes

1. Currently, waiting time during the PGT trajectory has been reduced significantly.
